# ASSESSMENT OF ACCURACY OF INCISIONAL AND NEEDLE BIOPSY IN SOFT-TISSUE TUMORS IN A BRAZILIAN CENTER OF REFERENCE

**DOI:** 10.1590/1413-785220253304e290125

**Published:** 2025-09-08

**Authors:** Bruna Canteri Delocco, Bruno de Oliveira Fiorelli, Igor Borges Abrantes, Eliane dos Santos Luz, Ana Cristina de Sá Lopes, Walter Meohas

**Affiliations:** 1 Instituto Nacional de Traumatologia e Ortopedia Jamil Haddad (INTO), Centro de Oncologia Ortopédica, Rio de Janeiro, RJ, Brazil.

**Keywords:** Soft Tissue Neoplasms, Biopsy, Diagnosis, Medical Oncology, Sarcoma, Tumores de Partes Moles, Biópsia, Diagnóstico, Oncologia, Sarcoma

## Abstract

**Objectives:**

To assess the accuracy of incisional biopsies (IB) and needle biopsies (NB) in soft-tissue tumors treated at a Brazilian center of reference, as well as the variables related to the demographic profile and treatment established.

**Methods:**

A retrospective, descriptive and observational study was conducted with patients with malignant soft tissue tumoral lesions, of indeterminate and intermediary malignancy, subjected to IB or NB at the institution from January 2010 to December 2019.

**Results:**

114 biopsies were performed in soft-tissue tumors of malignant lesions, of indeterminate and intermediary malignancy; of these 90 biopsies, 61 (67.7%) were IB and 29 (25.4%) were NB. It was necessary to perform a new collection in 5 cases among the NB (17.2%) and 1 case among the IB (1.6%). The IB accuracy was 83.6, while NB was 62.1. Of the 18 patients subjected to surgical treatment with radical margin, 7 were initially subjected to NB (36.8%) and 9 to IB (17.3%).

**Conclusion:**

Despite the advantages inherent to the percutaneous procedure of NB, IB must still be considered a diagnostic option in soft tissue tumors with high heterogeneity and degree of necrosis. ***Level of Evidence III; Diagnostic Studies - Investigating a Diagnostic Test***.

## RESUMO

**Objetivos:**

Avaliar a acurácia das biópsias incisionais (BI) e biópsias por agulha (BA) nos tumores de partes moles atendidos num centro de referência brasileiro, bem como as variáveis relacionadas ao perfil demográfico e tratamento instituído.

**Métodos:**

Foi realizado um estudo retrospectivo, descritivo e observacional, com pacientes com lesões tumorais de partes moles malignas, de malignidade indeterminada e intermediárias, submetidos a BI ou BA na instituição no período de janeiro de 2010 a dezembro de 2019.

**Resultados:**

Foram realizadas 114 biópsias de tumores de partes moles de lesões malignas, de malignidade indeterminada e intermediárias, destas 90 biópsias, 61 (67,8%) foram BI e 29 (25,4%) BA. Foi necessário nova coleta em 5 casos dentre as BA (17,2%) e 1 caso das BI (1,6%). A acurácia da BI foi de 83,6, enquanto da BA foi de 62,1. Dos 18 pacientes submetidos a tratamento cirúrgico com margem radical, 7 foram inicialmente submetidos à BA (36,8%) e 9 à BI (17,3%).

**Conclusão:**

Apesar das vantagens inerentes ao procedimento percutâneo da BA, a BI deve ainda ser considerada como opção diagnóstica em tumores de partes moles com alta heterogeneidade e grau de necrose. ***Nível de Evidência III; Estudo diagnóstico – Investigação de um exame para diagnóstico***.

## INTRODUCTION

Soft-part tumors can affect people of all ages, in the case of malignant lesions - sarcomas - most occur in adults from the 5th decade of life. The diagnosis of these lesions is carried out through clinical evaluation, imaging, biopsy and pathological analysis.

Biopsy is necessary to confirm the diagnosis and pathological analysis allows to determine the type and degree of the tumor. The techniques used to perform the diagnostic procedure can vary between the use of needle biopsy (NB) and incision biopsy (IB). A metaanalysis involving 17 studies showed that needle procedure is not inferior to IB in terms of defining the histopathological diagnosis.^1^ Both procedures have similar rates of complications, although NB causes more hematomas and equimosis, while IB is more responsible for operational wound infection and ulceration.

World literature data increasingly consolidates the NB as the gold standard in the investigation of soft-part tumors. IB has indications in precise cases, especially in places where the Jamshid needle is not available or when the heterogeneity of the tumor makes the histopathological diagnosis challenging.

For these reasons, it is important to analyze the data from a reference center in Brazil in musculoskeletal tumors to evaluate the reality in recent years in relation to incision and needle biopsies in the ability to define diagnosis and implications in the treatment of the patient with soft tissue tumors, which depend on tissue biopsy for histopathological diagnosis.^2^ The analysis of the accuracy of biopsies within these morbidities is of extreme importance for documentation of the epidemiological profile, evaluation of the results and repercussions with direct impact on treatment, on the type of surgery performed,^3,4^ and patient follow-up.^5,6^

Therefore, the aim of the study was to assess whether there is robust evidence regarding the best type of biopsy, in relation to diagnostic accuracy, in soft-part tumors treated in a reference center in oncological orthopedic treatment in Brazil.

## MATERIALS AND METHODS

This study was approved by the Institutional Ethics Committee (CAAE): 75396923.8.0000.5273). A retrospective, descriptive and observational study was conducted with patients with tumor lesions of soft parts who underwent biopsy at the institution from January 2010 to December 2019. The inclusion criteria were patients of any sex or age, with lesions of soft parts, in any anatomical location, submitted to IB or NB whose histopathological status is compatible with malignant lesions, of indeterminate or intermediate malignancy. Patients in whom the identified lesion was defined as metastasis, pseudotumoral lesion or benign lesion were excluded.

The data was collected, from the records, through the application of a check-list as a form of guidance for the information relevant to the research. After that they were inserted into the platform *Google Forms* and *Google Tabs* for the formulation of charts and tables for statistical development. The following data were collected: age, gender, ethnicity, type of initial biopsy, need for second biopsy and change of final histopathological status.

The software SPSS version 26 was used for data analysis, descriptive statistics were performed and the data were expressed as percentages. Student's t test was used to compare the variables.

## RESULTS

114 biopsies of tumors of soft parts of malignant, indeterminate and intermediate malignant lesions were performed in the period from January 2010 to December 2019 in the specialist care center in Orthopedic Oncology of a Brazilian orthopedic hospital.

The study population was predominantly male, white and older than 40 years of age (Table 1). The average diagnostic age of patients with soft-part tumor was 48.45 years, with a standard deviation of 20.94 years, the minimum being 03 years and the maximum 97 years. The sample fashion was unimodal (46 years), with a median of 50.5 years.

**Table 1:** Characterization of patients.

Variable	% (n)
**Sex**	
Female	52.6% (n = 60)
Male	47.4% (n = 54)
**Ethnicity**	
White	53.5% (n = 61)
Mixed race	32.5% (n = 37)
Black	16% (n = 14)
**Age group**	
Less than 20 years	13.2% (n = 15)
20 – 40 years	20.2% (n = 23)
40 – 60 years	33.3% (n = 38)
More than 60 years	33.3% (n = 38)

The anatomical location of these tumors was predominant in the lower limbs, 71.9% (n = 82), followed by the upper limbs with 22.8% (n = 26), trunk with 3.5% (n = 4), pelvis 0.9% (n = 1) and escapular waist 0.9% (n = 1).

Of the 114 biopsies performed during the study period, 10.5% (n = 12) were excisional biopsies (BE) and 10.5% (n = 12) were performed in other institutions and were therefore excluded from the study. After these initial exclusions there were 90 biopsies, 67.8% (n = 61) IB and 25.4% (n = 29) NB. Seven patients had to repeat the biopsy procedure at least once, of which one had been performed in another institution and was therefore excluded from the final accuracy analysis; five of these patients were submitted to a new IB, while the other two were submitted to BE.

In 46.5% of cases the histopathological study was sufficient to define the diagnosis, in the other 53.5% the immunohistochemistry was necessary to complete the diagnosis.

After the elaboration of the initial diagnosis, 81 patients had indication of surgical treatment as the first indication, the other 33 patients were referred to other services for some neoadjuvant therapy (radiotherapy or chemotherapy), of these patients, 14 were still undergoing surgical treatment at some point in this institution. Five patients who had a surgical proposal initially did not follow the treatment. Data on distribution, type of biopsy performed and proposed initial treatment are presented in Table 2.

**Table 2:** Distribution as to the type of biopsy, the need to repeat the biopsy and the treatment performed.

Variable	% (n)
**Type of initial biopsy**	
Needle	25.4% (n = 29)
Incision	67.8% (n = 61)
Excisional^1^	10.5% (n = 12)
Carried out at another institution^1^	10.5% (n = 12)
**Was it necessary to repeat the biopsy?**	
Yes	6.1% (n = 7)^2^
No	93.9% (n = 107)
**What technique was used in the second biopsy?**	
Incision	71.4% (n = 5)
Excisional	28.6% (n = 2)
**Technique used to define the diagnosis**	
Histopathological	46.5% (n = 53)
Immunohistochemical	53,5% (n = 61)
**Proposed initial treatment**	
Surgical	71.9% (n = 82)
Referred to another service^3^	28.1% (n = 32)

1 Patients referred to this service after performing the biopsy in another institution and patients undergoing BE were excluded from the study.

2 One of the biopsies that needed to be repeated was a biopsy performed in another institution and, therefore, was excluded from the final accuracy analysis.

3 Patients referred to treatment with chemotherapy or radiotherapy in another institution.

Of the needle biopsies, there was a need to perform a new collection in 5 cases, which represents 17.2% of cases. Of the incisions, only one had to be repeated, representing 1.6% of cases. In the NB cases that needed a new sample, IB was performed. There was only one case of IB that needed a new sample, was carried out then BE.

After resection of the tumor, there was a change in the final histopathological report (FHR) in 37.9% of the cases in which the first biopsy was carried out by needle, and in 16.4% of the patients in which IB was initially carried out. The results for both types of biopsies are contained in Table 3.

**Table 3:** Results relating to the type of biopsy performed, the need to repeat the biopsy and change of final histopathological record.

Variable	% (n)
**Type of biopsy** 1	
Incision biopsy	67.8% (n = 61)
Biopsy by needle	32.2% (n = 29)
**Unconcluding biopsies that needed to be repeated**	
Incision biopsy	1.6% (n = 1)
Biopsy by needle	17.2% (n = 5)
**Biopsies that had initial FHR altered**	
Incision biopsy	16.4% (n = 10)
Biopsy by needle	37.9% (n = 11)

1 After exclusion of excisional biopsies and biopsies performed in another institution.

After the analysis of the data exposed above, the accuracy was calculated from the total of samples that did not have the modified FHR divided by the total of the sample and multiplied by 100. The IB accuracy was 83.6, higher than the NB accuracy of 62.1. The accuracy data are shown in Table 4.

**Table 4:** Accuracy of incision and needle biopsies performed in the period from January/2010 to December 2019.

Accuracy	%
**Type of biopsy**	
Incision biopsy	83.6%
Biopsy by needle	62.1%

Of the total of 90 biopsies evaluated in this study, 61 incision and 29 per needle, 71 were submitted to surgical treatment at some point, of which 71, 19 were NB and 52 IB. Evaluating the type of surgery proposed and the final outcome of the patient, of these 71 biopsies submitted to surgical treatment at some point, in 18 was performed radical treatment, when the entire compartment where the tumor is located is resecated, e.g. amputation or desarticulation of limb.

Thus, of the 18 patients undergoing radical surgical treatment, 7 initially underwent NB, which implies that 36.8% of the NB patients undergoing surgical treatment needed surgery with radical margin. Regarding IB, 9 of the 52 biopsies undergoing surgical treatment at some point required treatment with radical margin, which corresponds to 17.3%.

## DISCUSSION

This study showed that the accuracy of the IB was higher than of the NB, in addition, approximately 40% of the NB presented discrepancy in the final diagnosis.

Biopsy for the histological definition of soft-part tumors is a crucial part of the correct management and treatment of these patients, as an inaccurate biopsy delays the start of the optimal treatment and an incorrect biopsy compromises the entire sequential treatment proposed to the patient.

One study compared ultrasound-guided tumor biopsies of incisional and needle soft parts and found an accuracy for the IB of 100%, very similar to this study, which found 96.7%.^7^ For the ultrasound-guided NB, this also found an accuracy of 100%, while in our study the accuracy for the NB was 55.2%. This significant difference between the accuracy found can be explained because in the study period we did not use any auxiliary imaging method in the NB, such as ultrasound.

Published data indicate in tumors of malignant soft parts, such as sarcomas, a rate of diagnostic inaccuracy ranging from 20 to 30%.^8,9^ This is due to the large heterogeneity of these tumors. In this study, the diagnostic inaccuracy was less, about 6%.

Survival of patients with soft-part sarcoma varies considerably based on factors such as the location of the tumor and the stage of the disease at the time of diagnosis, highlighting the importance of early detection and appropriate treatment to improve survival chances in patients with soft-part sarcoma.

This study evaluated a very representative number of patients for being rare malignant tumors in an extensive period of analysis. Thus, this study shows the current scenario of a reference center in Brazil with a large volume of tumors treated per year. However, when compared with general orthopedic pathologies the number of patients evaluated is small, and there is always a limitation of this type of study. Another limitation of this study is that the biopsies that needed to be repeated were repeated as IB or BE, none per NB.

New analyses may complement the findings of this study with the comparative evaluation of the results obtained with the NB of ultrasound-guided soft parts.

## CONCLUSION

Our findings show that despite the advantages inherent in the percutaneous procedure of NB, such as faster procedure, shorter hospital time and less invasion of the patient, IB should still be considered as an option, especially in tumors of soft parts with high heterogeneity and degree of necrosis inside.

## References

[1] Birgin E, Yang C, Hetjens S, Reissfelder C, Hohenberger P, Rahbari NN (2020). Core needle biopsy versus incisional biopsy for differentiation of soft-tissue sarcomas: A systematic review and meta-analysis. Cancer.

[2] Agulnik M, Bui MM, Carr-Ascher J, Choy E, Dry S, Ganjoo KN NCCN. Guidelines Version 2.2022 Soft Tissue Sarcoma NCCN Evidence Blocks TM.[Internet].

[3] Sbaraglia M, Bellan E, Dei Tos AP (2021). The 2020 WHO Classification of Soft Tissue Tumours: news and perspectives. Pathologica.

[4] Steffner RJ, Jang ES (2018). Staging of Bone and Soft-tissue Sarcomas. J Am Acad Orthop Surg.

[5] Morita SY (2018). Surgical management of truncal soft tissue sarcoma and other selected soft tissue neoplasms. Chin Clin Oncol.

[6] Alamanda VK, Crosby SN, Archer KR, Song Y, Schwartz HS, Holt GE (2013). Predictors and clinical significance of local recurrence in extremity soft tissue sarcoma. Acta Oncol.

[7] Pazourek L, StaniczkovÁ Zambo I, TomÁŠ T, Mahdal M (2020). [Open Incisional Biopsy and Ultrasound-Guided Core Needle Biopsy of Extremity-Localized Musculoskeletal Tumors]. Acta Chir Orthop Traumatol Cech.

[8] Sbaraglia M, Dei Tos AP (2019). The pathology of soft tissue sarcomas. Radiol Med.

[9] Thway K, Wang J, Mubako T, Fisher C (2014;). Histopathological diagnostic discrepancies in soft tissue tumours referred to a specialist centre: reassessment in the era of ancillary molecular diagnosis. Sarcoma.

